# Gastric defect closure with reopenable clip over-the-line method and dual-action tissue clip for 3-point fixation

**DOI:** 10.1055/a-2489-8253

**Published:** 2024-12-10

**Authors:** Tatsuma Nomura, Takanobu Mitani, Yuto Ikadai, Hiroaki Kumazawa, Yoshiaki Isono, Makoto Kobayashi, Katsumi Mukai

**Affiliations:** 1 Department of Gastroenterology, Suzuka General Hospital, Suzuka, Japan; 2 Department of Endoscopy Center, Suzuka General Hospital, Suzuka, Japan; 3 Department of Gastroenterology, Yokkaichi Municipal Hospital, Yokkaichi, Japan

After endoscopic submucosal dissection (ESD) of the stomach, defect closure is difficult because a submucosal dead space (SDS) is created by the thick muscle layer and mucosa, even with the use of ordinary clips alone 1 . Therefore, we devised the reopenable clip over-the-line method (ROLM), which enables the amount of SDS to be reduced 2 3 4 . However, this method is time consuming. Therefore, we further devised a method that uses ROLM and the dual-action tissue (DAT) clip for 3-point fixation for defect closure ( Fig. 1 , Video 1 ).

**Fig. 1:**
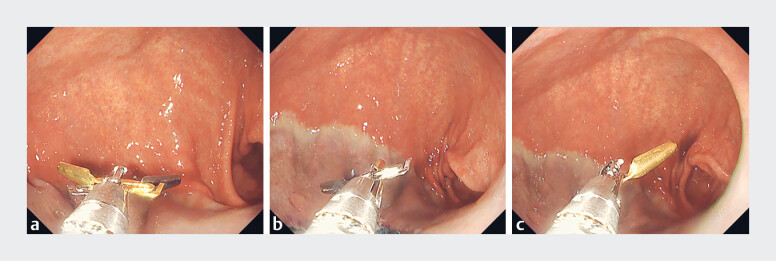
Through-the-scope dual-action tissue (DAT) clip. **a** Both clips with their teeth open. **b** The silver clip with teeth open. **c** The gold clip with teeth open.

Defect closure after gastric endoscopic submucosal dissection using the reopenable clip over-the line method and a dual-action tissue clip for 3-point fixation.Video 1

Here, we describe the case of a 68-year-old man with early cancer in the posterior wall of the middle gastric body ( Fig. 2 ). A 40-mm mucosal defect occurred after ESD, which was closed using ROLM with 3-point fixation. First, the defect edge was grasped using the first tooth of the DAT clip. Then, the contralateral defect edge, and if possible, the submucosal layer above the muscle layer in the middle of the defect were grasped using the second tooth of the clip. After confirming that the edges of the defect on both sides were fixed, a clip was placed. The first clip, with a line for ROLM, was then placed on the most distal defect edge. The line was threaded through the holes in the reopenable-clip teeth. Subsequently, a clip with a line threaded through one tooth was placed at the contralateral edge of the defect. The defect edges were already in close proximity; thus, the time required for additional clips was reduced.

**Fig. 2:**
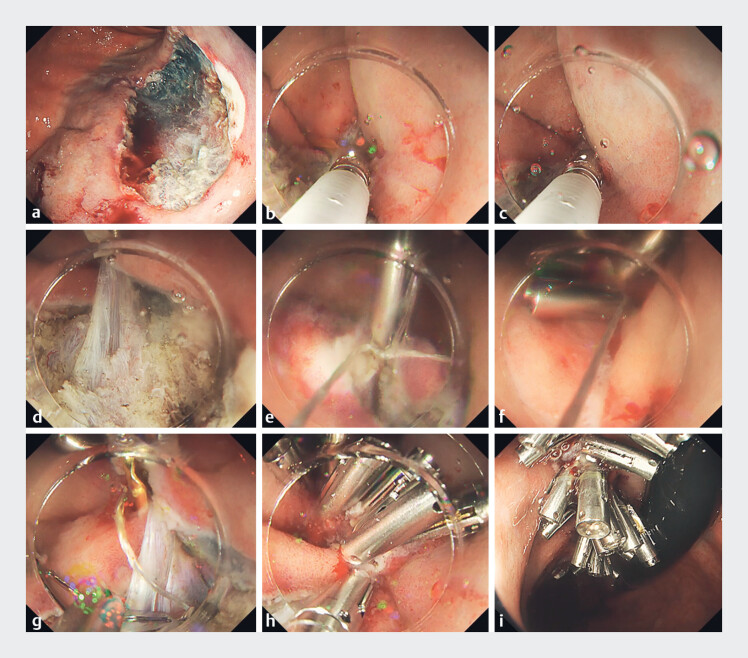
Closure of a 40-mm defect after gastric endoscopic submucosal dissection (ESD) using the reopenable clip over-the-line method (ROLM) and a dual-action tissue (DAT) clip for 3-point fixation. **a** The 40-mm post-ESD defect on the posterior wall of the gastric body. **b** One edge of the defect was grasped by the DAT clip tooth. **c** The contralateral edge of the defect and the submucosa above the muscle layer were then grasped by the second tooth of the clip. **d** The defect edges on both sides and the muscle layer and submucosa above it were grasped together using the DAT clip. **e** The first clip with line was then placed on the distal mucosal defect edge. **f** The mucosal defect was straightened for the DAT clip, so that the reopenable clip with a line through the tooth on one side could be placed easily. **g** ROLM was performed without embedding the DAT clip. **h** Reopenable clips with a line through the tooth on one side were repeatedly placed on the defect edge. **i** Complete closure of the gastric post-ESD defect after ROLM with 3-point fixation.

ROLM allows the placement of clips without embedding the through-the-scope DAT clip on the defect side. The defect was completely closed by repeatedly placing clips on the defect edge on one side. A total of 22 reopenable clips were used, and the time required for ROLM was only 12 minutes.

Endoscopy_UCTN_Code_TTT_1AO_2AO

## Notes

Endoscopy E-Videos https://eref.thieme.de/e-videos
*E-Videos* is an open access online section of the journal *Endoscopy* , reporting on interesting cases and new techniques in gastroenterological endoscopy. All papers include a high-quality video and are published with a Creative Commons CC-BY license. Endoscopy E-Videos qualify for HINARI discounts and waivers and eligibility is automatically checked during the submission process. We grant 100% waivers to articles whose corresponding authors are based in Group A countries and 50% waivers to those who are based in Group B countries as classified by Research4Life (see: https://www.research4life.org/access/eligibility/ ). This section has its own submission website at https://mc.manuscriptcentral.com/e-videos .
